# Association between increased screen time during the COVID-19 pandemic and changes in alcohol use behaviours among Canadian adolescents: a prospective cohort study

**DOI:** 10.24095/hpcdp.45.11/12.03

**Published:** 2025

**Authors:** Thepikaa Varatharajan, Christa Orchard, Erin Collins, Ahmed Al-Jaishi, Salah Uddin Khan, Kate Battista, Scott T. Leatherdale, Rojiemiahd Edjoc

**Affiliations:** 1 School of Public Health Sciences, University of Waterloo, Waterloo, Ontario, Canada; 2 Centre for Surveillance and Applied Research, Health Promotion and Chronic Disease Prevention Branch, Public Health Agency of Canada, Ottawa, Ontario, Canada; 3 Dalla Lana School of Public Health, University of Toronto, Toronto, Ontario, Canada; 4 School of Epidemiology and Public Health, University of Ottawa, Ottawa, Ontario, Canada

**Keywords:** youth, alcohol, pandemic, screen time, social media

## Abstract

**Introduction:**

The aim of this study was to investigate the association between increased screen time and changes in alcohol use among adolescents in Canada during the COVID-19 pandemic.

**Methods:**

Self-reported data were retrieved from secondary school students who participated in the COMPASS study pre-pandemic (T1, the 2018 to 2019 school year) and at least once after the onset of the pandemic, at T2, in May to July 2020, and/or at T3, the 2020 to 2021 school year. We used multinomial logistic regression models to estimate the adjusted odds ratio (aOR) of association between change in screen time since the pandemic onset with change in alcohol use at T2 and T3.

**Results:**

A large majority of the sample, aged 12 to 19 years, reported increased screen time (92% at T2, and 91% at T3) since the pandemic onset. Increased screen time was associated with higher odds of alcohol use initiation during T2 (aOR = 1.66; 95% confidence interval [CI]: 1.09–2.51) and T3 (aOR = 1.45; 95% CI: 1.22–1.73). Among students who used alcohol at baseline, increased screen time (aOR = 0.55; 95% CI: 0.40–0.75) and social media use (aOR = 0.72; 95% CI: 0.59–0.87) were linked to decreased odds of reduction compared to maintenance in drinking frequency at T3.

**Conclusion:**

We identified a relationship between increased screen time and initiation of and change in alcohol use during the pandemic among Canadian adolescents. Future research could investigate why this relationship exists and identify at-risk groups and potential interventions that prevent and reduce drinking in adolescents.

**Highlights**

- About 91% of the surveyed adolescents reported that they had increased their screen time since the onset of the pandemic, both early in the pandemic, in May to July 2020, and later, during the 2020 to 2021 school year.
- Increased screen time was associated with higher odds of adolescents initiating alcohol use, particularly during the early part of the pandemic, in May to July 2020 (adjusted odds ratio [aOR] = 1.66; 95% confidence interval [CI]: 1.09–2.51), but also during the 2020 to 2021 school year (aOR = 1.45; 95% CI: 1.22–1.73).
- Among the adolescents who used alcohol at baseline, increased screen time (aOR = 0.55; 95% CI: 0.40– 0.75) and social media use (aOR = 0.72; 95% CI: 0.59–0.87) were linked with lower odds of reduction compared to maintenance in drinking frequency.

## Introduction

Screens (e.g. cellphones, tablets, computers and TVs) are integrated into the everyday lives of Canadian children and adolescents, at home and in school.^1^,^2^ Screen time refers to any time spent with an electronic device; such time may be active (i.e. cognitively and/or physically engaging in screen-based activities) or passive (i.e. engaging in sedentary screen-based activities and/or passively receiving screen-based information).^3^-^5^ Examples of active screen time include virtual reality fitness games, video calls and chatting online, while passive screen time activities include watching TV or scrolling through social media apps (defined as any online interactive platform in which individuals can create and share user-generated content in the form of pictures, personal messages, videos, etc.).^3^-^6^

The Canadian 24-hour Movement Guidelines (24-HMGs) recommend no more than 2hours per day of recreational screen time for children and youth aged 5 to 17years.^7^ Emerging evidence shows excessive screen time to be a prominent independent risk factor for various adverse physical, cognitive, behavioural and mental health outcomes among adolescents, including sedentary behaviour, social isolation, increased risk of internalizing problems (e.g. symptoms of depression and anxiety), lower self-control and poor sleep.^2^,^8^

In 2018 to 2019, about 31% of Canadian youth aged 12 to 17 years spent an average of 3.8 hours per day on screens, relatively unchanged from the average daily screen time of North American youth in the previous decade.^9^,^10^ With the onset of the COVID-19 pandemic and the implementation of public health measures to contain spread of infection (e.g. school closures, physical distancing, travel restrictions and home confinement), adolescents’ screen time increased.^4^,^5^,^11^-^16^ Three-quarters of the Canadian parents (78.8%) responding to the 2020 ParticipACTION survey (n=1472) reported that their children’s (5–17 years) screen time had increased over the one month after the World Health Organization declared COVID-19 a global pandemic and during the height of restrictions.^17^ Canadian adolescents (14–17 years; n = 774) spent approximately 4.21 hours per day on recreational screen time.^18^ Moore et al. reported that more children and youth met screen time recommendations during the second wave of the pandemic (October 2020; 25%) than during the first wave (April 2020; 11.3%).^19^

Excessive screen time may also vary, particularly among adolescents, depending on the type of media used (e.g. TV, social media, Internet, video games).^20^,^21^,^22^ In addition, screen preferences may shift with age, for example, the time spent on traditional media devices (e.g. watching TV and playing video games) may remain the same, while posting and scrolling through social media increases and peaks from mid- to late adolescence.^20^,^23^ In 2025, about 70% of adolescents in the United States (n = 10092; 11–15 years) reported having at least one social media account,^21^ with some youth spending more than 3hours per day on social media platforms, most commonly, YouTube, Snapchat, Instagram and Tiktok.^6^,^8^,^19^,^24^

A noted consequence of excessive screen time during the pandemic were parallel changes in individuals’ drinking behaviours.^5^,^25^,^26^ These findings, which align with pre-pandemic research, show cross-sectional and prospective associations between screen time and substance use including alcohol, cannabis and tobacco use.^27^-^30^ In 2021, Tebar et al. reported a positive association between increased television time and the desire to drink and a negative association between increased computer use and alcohol consumption in a sample of Brazilian adults.^27^ Su et al. reported a lower likelihood of alcohol use among Chinese students (5–17 years) who only adhered to the 2-hour daily screen time guideline compared to peers who met none of the 24-HMGs.^26^ Wiciak et al. observed that young adults’ average weekly screen times increased for entertainment by 8.0 hours and for social media use by 6.8 hours during the pandemic.^24^ The excessive entertainment-related screen time was significantly associated with higher levels of alcohol abuse and more servings of alcohol per week.^24^

Since most of the existing evidence used a cross-sectional design or focused on adult populations, our aim was to assess the association between self-reported increased levels of screen time, in response to the COVID-19 pandemic, and changes in alcohol use (initiated, escalated or reduced) among adolescents. Taking into account that social media use is popular at this age,^6^,^8^,^21^,^31^ a secondary objective of our study was to determine the direction and magnitude of the association between self-reported changes in social media use and alcohol use during the pandemic.

## Methods

***Ethics approval***

All COMPASS procedures were approved by the University of Waterloo Office of Research Ethics (ORE# 30118), CIUSSS de la Capitale-Nationale–Universit Laval (#MP-13-2017-1264) and participating school boards.

***Data source***

We report this study in accordance with STROBE guidelines.^32^ The data source for this study is COMPASS (Cannabis, Obesity, Mental health, Physical Activity, Sedentary behaviour and Smoking), a longitudinal cohort study of students attending schools in British Columbia, Ontario and Quebec. The COMPASS study recruits a convenience sample of students on a rolling basis from Grades 9 through 12 (Secondary I–V in Quebec; 12–19 years) and is conducted annually.^33^ (A complete description of COMPASS study methods is available at https://uwaterloo.ca/compass-system/).

***Design and participants***

We used student-level data from COMPASS waves 7 (T1: the 2018–2019 school year), 8 (T2: the 2019–2020 school year) and 9 (T3: the 2020–2021 school year). Included were those students who completed the baseline (T1) questionnaire and at least one follow-up questionnaire at T2 or T3. At T1, student data were collected via a paper-based questionnaire completed in-person and during class time.^34^ In response to the school closures, COMPASS switched to using an online questionnaire, which was emailed to all students by their schools.^34^ Student data at T2 (from May to July 2020) and T3 were collected online using the Qualtrics XM survey software (Qualtrics, Provo, UT, US). As the pre- and early pandemic periods overlapped with the 2019 to 2020 school year, participants who completed the questionnaire in T2 prior to the onset of the pandemic in March 2020 were excluded. Therefore, only those who completed a questionnaire between May and July 2020 were included in the T2 sample, resulting in a smaller cohort of participants than in T1 and T3.

***Measures***

**Primary exposure variable: Increased screen time since COVID-19 pandemic onset**

In T2 (May to July 2020) and T3 (September 2020 to June 2021), students were asked whether their “time spent communicating with friends online,” “time spent watching TV/movies or playing video games” and “time spent surfing/posting on social media” had “increased,” “stayed the same” or “decreased” after the onset of the pandemic. A binary indicator was used to indicate whether students reported that their use of any of these three activities had increased or that all three types of screen time had decreased or stayed the same (or the question was not applicable).

**Secondary exposure: Increased social media use since COVID-19 pandemic onset**

Change in social media use since the pandemic onset was based on students’ responses only to the activity “time spent surfing/posting on social media.” Responses were collapsed into a binary variable, “increased” or “decreased/stayed the same.”

**Outcome: Change in alcohol use**

At all three time points, respondents were asked to rate their frequency of alcohol use with the following response options: “I have never drunk alcohol,” “I did not drink alcohol in the last 12 months,” “I have only had a sip of alcohol,” “less than once a month,” “once a month,” “2 or 3times a month,” “once a week,” “2 or 3times a week,” “4 to 6 times a week” and “every day.” We compared T1 with T2 responses and T1 with T3 responses, with two outcome variables captured for two different groups: (1) initiation versus abstinence, and (2) escalation or reduction versus maintenance.

*Outcome 1: Initiation versus abstinence*

We determined whether students who reported no alcohol use in the past year at T1 (indicated by the responses “I have never drunk alcohol,” “I did not drink alcohol in the last 12 months” or “I have only had a sip of alcohol”) reported no use at the time of follow-up (i.e. abstinence) or initiation of alcohol use at the time of follow-up.

*Outcome 2: Escalation, maintenance and reduction*

We determined whether the frequency of alcohol use among students who reported at least some alcohol use at T1 had increased (i.e. escalation), decreased (i.e. reduction) or remained the same (i.e. maintenance), using the same groupings as in prior studies with this cohort.^35^

***Covariates***

Covariates were selected a priori based on existing research^13^,^20^,^36^ and expected conceptual relationships. Characteristics captured at baseline included age, sex and ethnicity, because of the known differences in screen time and alcohol use across these sociodemographic groups.^36^ Given the correlation between mental health, screen time and alcohol use, including during the pandemic,^13^,^20^ we assessed for symptoms of depression using the Centre for Epidemiologic Studies Depression Revised 10-item (CESD-R-10) scale and for symptoms of anxiety using the Generalized Anxiety Disorder 7-item (GAD-7) scale. Both scales have been validated for use among adolescents.^37^,^38^

We captured frequency of alcohol use at baseline as well as self-reported past-month cigarette and e-cigarette use, past-year cannabis use and past-year nonmedical prescription opioid use (i.e. oxycodone, fentanyl or other pain killers). Student-level measures of alcohol and other substance use are consistent with national surveillance tools for youth populations.^39^ We computed average screen time in minutes per day by summing self-reported minutes spent watching TV, playing video games, surfing the Internet and messaging. Earlier evaluations indicated one-week test–retest intraclass correlation coefficients from 0.54 to 0.86 for each item.^10^,^40^,^41^

***Analyses***

Using multinomial logistic regression models, we examined the association between change in alcohol use and change in overall screen time and social media use. The T2 cohort was divided into those who reported drinking alcohol at baseline and those who did not. Models were then run to examine whether increases in screen time and social media use were associated with the odds of initiation of alcohol use among students who did not drink alcohol and changes in the odds of escalation or reduction in alcohol use among students who did. This was repeated for the T3 cohort, resulting in eight separate models for the two different exposures at two time points per baseline group (those who drank alcohol and those who did not).

All regression models adjusted for school-level clustering, province of school location, sociodemographic characteristics, mental health status, substance use and average daily minutes of screen time at baseline. Models of reported drinking at baseline were also adjusted for baseline alcohol use frequency, that is, either occasional alcohol use (once a month or less frequently) or regular alcohol use (2 or 3 or more times a month). Beta estimates from the models were exponentiated to obtain crude and adjusted odds ratios with corresponding 95% confidence intervals.

Where there was item-level missingness on the mental health scales, we included all individuals who reported at least one item on a scale, using the mean of non-missing responses to estimate the overall score on the scale.^42^ Where responses on input variables were missing, we conducted a complete case analysis, excluding individuals with missingness. As a sensitivity analysis, we repeated the main analysis removing social media from the measure of screen time, to examine whether social media use was driving the direction or size of the effects.

Analyses were completed using RStudio version 4.2.1, using the nnet package to run the multinomial regression models.^43^

## Results

***Sample***

Altogether 14865 students at the schools taking part in all three data collection waves completed the T1 baseline questionnaire in the 2018 to 2019 school year and at least one follow-up questionnaire between May 2020 and June 2021. A total of 4103 students completed the T2 questionnaire in May to July 2020 (27.0% of students in schools participating in COMPASS wave 8), 12648 completed the T3 questionnaire in the 2020 to 2021 school year (85.0% of students in schools participating in COMPASS wave 9) and 1886 completed both T2 and T3 questionnaires (12.7% of students in schools participating in COMPASS waves 8 and 9). To reduce sample bias during the period immediately after the implementation of pandemic-related restrictions (i.e. T2), only those schools that participated in at least one follow-up period were included.

After removing students with missing exposure and outcome data, the final sample sizes were 3419 (83.3%) for students completing both T1 and T2 questionnaires and 10770 (85.2%) for students completing both T1 and T3 questionnaires. (A flowchart illustrating study participant inclusions and exclusions is available upon request from the authors.)

The variables with a large number of nonresponses included change in screen time and in social media use, for which about 12% (n=501 and n=503, respectively) of respondents were missing data at T2 and about 9% (n=1157 and n=1164, respectively) at T3. In addition, 8.4% (n=344) of those responding to the T1 and T2 questionnaires and 7% (n=882) of those responding to the T1 and T3 questionnaires were missing data on change in alcohol use. (A comparison of respondents with and without missing data for each follow-up period is available upon request from the authors.)

***Baseline characteristics***

More than half of the respondents were female, more than three-quarters self-identified as White and most were 15years old or younger. While mean CESD-R-10 and GAD-7 scale scores were less than the diagnostic cut-off scores of 10, 31% of the T2 respondents met the criteria for a depressive disorder and 21% for an anxiety disorder. At 25% and 16%, respectively, these proportions were slightly lower at T3 (Table 1).

**Table 1:** Characteristics of students participating in the COMPASS study at baseline (2018–2019 school year) and at least one follow-up time point in 2020 or 2021, Canada

Characteristics	T1 + T2 respondents (n = 3419)^a^	T1 + T3 respondents (n = 10 770)^b^
Sex, n (%)		
Female	2215 (64.8)	6284 (58.3)
Male	1204 (35.2)	4486 (41.7)
Age in years, n (%)		
12	252 (7.4)	1354 (12.6)
13	598 (17.5)	2608 (24.2)
14	847 (24.8)	3774 (35.0)
15	953 (27.9)	2594 (24.1)
16	606 (17.7)	418 (3.9)
17–19	163 (4.8)	22 (0.2)
Ethnicity, n (%)		
White	2645 (77.4)	8691 (80.7)
Black	105 (3.1)	205 (1.9)
Asian	239 (7.0)	550 (5.1)
Latin American	55 (1.6)	160 (1.5)
Other/mixed	375 (11.0)	1164 (10.8)
Mental health scale, mean score (IQR)		
CESD-R-10^c^	6.0 (3.0, 11.0)	6.0 (3.0, 10.0)
GAD-7^d^	4.0 (2.0, 8.0)	3.0 (1.0, 7.0)
Substance use, n (%)		
Past-month cigarette use	111 (3.2)	224 (2.1)
Past-month e-cigarette use	654 (19.1)	1721 (16.0)
Past-year cannabis use	368 (10.8)	809 (7.5)
Past-year nonmedical prescription opioid use	114 (3.3)	351 (3.3)
Mean daily screen time, minutes (IQR)		
Total	300 (210, 450)	285 (180, 420)
TV	90 (60, 150)	90 (45, 135)
Gaming	15 (0, 90)	30 (0, 120)
Internet	60 (30, 150)	60 (30, 120)
Messaging	45 (15, 120)	30 (15, 90)
Alcohol drinking frequency, n (%)		
None^e^/only a sip	2015 (58.9)	6957 (64.8)
Less than once a month	670 (19.6)	1896 (17.6)
Once a month	288 (8.4)	738 (6.9)
2 or 3 times a month	312 (9.1)	817 (7.6)
Once a week	84 (2.5)	218 (2.0)
2 or 3 times a week	36 (1.1)	94 (0.9)
4 to 6 times a week	11 (0.3)	17 (0.2)
Every day	3 (0.1)	15 (0.1)

**Abbreviations: **CESD-R-10, Centre for Epidemiologic Studies Depression Revised 10-item scale; GAD-7, Generalized Anxiety Disorder 7-item scale; IQR, interquartile range. 

^a^ Respondents who completed the COMPASS questionnaire at T1 (2018–2019 school year, or “pre-pandemic period”) and T2 (May to July 2020, or “early pandemic period”). 

^b^ Respondents who completed the COMPASS questionnaire at T1 (2018–2019 school year, or “pre-pandemic period”) and T3 (September 2020 to June 2021, or “late pandemic period”). 

^c^ CESD-R-10 scores range from 0 (lowest) to 30 (highest). 

^d^ GAD-7 scores range from 0 (lowest) to 21 (highest). 

^e^ Made up of the response options “I have never drunk alcohol” and “I did not drink alcohol in the last 12 months.” 

Past-month cigarette use and past-year nonmedical prescription opioid use were relatively uncommon, but 19% of respondents at T2 and 16% at T3 reported using e-cigarettes in the past month. A little more than one-third reported drinking alcohol at baseline, and average daily screen time was 300 minutes (5 hours) at T2 and 285 minutes (4.75 hours) at T3 (Table 1).

***Changes in screen time and alcohol use***

Most of the respondents (92% at T2 and 91% at T3) reported increasing their screen time since the onset of the pandemic (Table 2). About two-thirds (69%) reported increased social media use at T2 and T3. A larger proportion of female students than of male students reported increased screen time at T3 (92.6% vs. 88.6%; *p*<0.001). No significant differences between females and males were observed at T2. A larger proportion of female students than of male students reported increased time on social media at T2 (73% vs. 61%; *p*< 0.001) and at T3 (74% vs. 60%; *p*<0.001).

**Table 2:** Changes in screen time and alcohol use among students participating in the COMPASS study, from baseline (2018–2019 school year) to at least one follow-up time point (in 2020 or 2021), Canada

Change in behaviour	T1 + T2 respondents (n = 3419)^a^	T1 + T3 respondents (n = 10 770)^b^
Screen time use, n (%)		
No increase/decrease	260 (7.6)	978 (9.1)
Increase	3159 (92.4)	9792 (90.9)
Social media use, n (%)		
No increase/decrease	1071 (31.3)	3391 (31.5)
Increase	2348 (68.7)	7379 (68.5)
Change in alcohol use, n (%)		
No alcohol use at baseline^c^		
Abstinence^d^	1462 (72.6)	3746 (53.7)
Initiation^e^	553 (27.4)	3229 (46.3)
Alcohol use at baseline^c^		
Escalation^f^	523 (37.3)	1661 (43.8)
Maintenance^g^	506 (36.0)	1117 (29.4)
Reduction^h^	375 (26.7)	1017 (26.8)

^a^ Respondents who completed the COMPASS questionnaire at T1 (2018–2019 school year, or “pre-pandemic period”) and T2 (May to July 2020, or “early pandemic period”). 

^b^ Respondents who completed the COMPASS questionnaire at T1 (2018–2019 school year, or “pre-pandemic period”) and T3 (September 2020 to June 2021, or “late pandemic period”). 

^c^ Percentages are based on the total number of participants who did not use alcohol (n = 2015) or participants who used alcohol (n = 1404) at baseline. 

^d^ No alcohol use at the time of follow-up. 

^e^ Alcohol use had been initiated at the time of follow-up. 

^f ^ Frequency of alcohol use had increased. 

^g^ Frequency of alcohol use remained the same. 

^h^ Frequency of alcohol use decreased. 

Of those respondents who did not drink alcohol at baseline, 27% had initiated drinking by T2 and 46% by T3 (Table 2). Of those students who reported drinking at baseline, 36% maintained, 37% escalated and 27% reduced their drinking frequency at T2; and 29% maintained, 44% escalated and 27% reduced their drinking frequency at T3. 

A larger proportion of female than of male students initiated drinking at T2 (68% vs. 32%) and T3 (59% vs. 41%) (*p*<0.001). Differences between females and males were observed for students who reduced their drinking at T2 (65% females vs. 35% males; *p*=0.03) and those who escalated their drinking at T3 (58% females vs. 42% males; *p*<0.001). (A breakdown of changes in screen time and alcohol use by sex is available upon request from the authors.)

***Changes in alcohol use***

Among students who did not use alcohol at baseline, an increase in screen time and social media use, respectively, was linked to a 66% and 69% increase in the adjusted odds of initiated alcohol use at T2 and a 45% and 75% increase in the adjusted odds of initiated alcohol use at T3 (Table 3).

**Table 3:** Association between changes in screen time and initiation of or change in alcohol use among students participating in the COMPASS study at time points in 2020 or 2021, Canada

Change in behaviour	T2 vs. T1 responses (n = 3419)^a^ OR (95% CL)			T3 vs. T1 responses (n = 10 770)^b^ OR (95% CL)		
	Initiation vs. abstinence	Escalation vs. maintenance	Reduction vs. maintenance	Initiation vs. abstinence	Escalation vs. maintenance	Reduction vs. maintenance
Increased screen time						
Unadjusted	1.54 (1.03, 2.30)	1.17 (0.72, 1.89)	1.01 (0.61, 1.67)	1.48 (1.25, 1.76)	0.73 (0.54, 0.97)	0.55 (0.40, 0.74)
Adjusted^c^	1.66 (1.09, 2.51)	1.17 (0.72, 1.91)	1.02 (0.60, 1.72)	1.45 (1.22, 1.73)	0.76 (0.56, 1.03)	0.55 (0.40, 0.75)
Increased social media use						
Unadjusted	1.77 (1.42, 2.20)	1.34 (1.00, 1.79)	0.85 (0.63, 1.15)	1.78 (1.61, 1.98)	1.02 (0.86, 1.22)	0.75 (0.55, 0.94)
Adjusted^c^	1.69 (1.34, 2.12)	1.31 (0.97, 1.77)	0.88 (0.56, 1.19)	1.75 (1.57, 1.95)	1.08 (0.90, 1.30)	0.72 (0.59, 0.87)

**Abbreviations: **CESD-R-10, Centre for Epidemiologic Studies Depression Revised 10-item scale; CL, confidence limit; GAD-7, Generalized Anxiety Disorder 7-item scale; OR, odds ratio. 

^a^ Completed COMPASS questionnaire responses at T2 (May to July 2020, or “early pandemic period”) vs. T1 (2018–2019 school year, or “pre-pandemic period”). 

^b^ Completed COMPASS questionnaire responses at T3 (September 2020 to June 2021, or “late pandemic period”) vs. T1 (2018–2019 school year, or “pre-pandemic period”). 

^c ^Adjusted for baseline (T1, the 2018–2019 school year) covariates: sex, age, ethnicity, school location province, CESD-R-10 score, GAD-7 score, cigarette use, e-cigarette use, cannabis use, nonmedical prescription opioid use, average daily minutes of screen time, and among students that drink alcohol, frequency of alcohol consumption. 

Among participants who used alcohol at baseline, increases in screen time and social media use in the adjusted models were not associated with escalation of alcohol use compared to maintenance of baseline drinking frequency, at either T2 or T3. At T2, increases in screen time had no significant effect on the adjusted odds of reducing versus maintaining pre-pandemic drinking frequency. However, at T3, increases in screen time and social media were associated with a 55% and 72% decrease in the adjusted odds of reducing versus maintaining alcohol use, respectively.

When social media use was excluded from the measure of screen time in the analysis, we found the identified effects to be similar but slightly smaller, indicating that the association is not solely due to social media–related screen time. (Results from this analysis are available upon request from the authors.)

## Discussion

The aim of this study was to identify the association between increased screen time during the COVID-19 pandemic and changes in alcohol use among adolescents in Canada. We found that increased screen time was linked to the initiation of alcohol use during the COVID-19 pandemic. Among those adolescents who drank alcohol prior to the onset of the pandemic, increases in social media use were linked to lower odds of reducing alcohol use by T3 (September 2020 to June 2021).

Our findings add to prior research that identified a link between alcohol use and both social media use and other types of screen use during the COVID-19 pandemic.^27^,^29^,^30^ Of note, we demonstrated that the link between increased social media use and initiating alcohol use persisted even when in-person interactions were restricted during the pandemic.

The potential reasons for this link are complex. One reason could be increased exposure to alcohol-related content, including advertisements, marketing and peer- or influencer-generated content.^21^,^30^,^44^ Other posited reasons include the relationship between screen time and adolescent brain development, with changes in the reward system potentially influencing drinking behaviour.^45^ Screen time and social media use have also been linked to poor mental health, which may have a mediating effect on coping strategies such as alcohol use.^46^ Given the impact of the pandemic on mental health, any such mediating relationship warrants further attention.

While we found evidence for an association between increased screen time and initiation of alcohol use by respondents who did not drink alcohol before the onset of the pandemic, the evidence for change in alcohol use among those who already did was less clear. Increased social media use appeared to be linked to reductions in alcohol use early and later in the pandemic (i.e. at T2 and at T3). Later in the pandemic, increases in screen time continued to have no effect on escalation of use, but did lower the odds of reducing alcohol use.

The reasons why increased screen time differentially affected participants who did not use alcohol compared to those who did are unclear. Possible explanations could include students’ reduced ability to access larger quantities of alcohol during the pandemic and changing motives for using alcohol (e.g. coping motives, conformity motives, social and enhancement motives).^47^ Further research is required to examine these relationships and to determine whether those who initiated alcohol use during the pandemic continued drinking after the pandemic.

***Strengths and limitations***

Several limitations of this study warrant consideration. First, students were included if their schools participated in the COMPASS study in the 2018 to 2019 school year and in either the May to July 2020 or the 2020 to 2021 school years or both periods. While this may have excluded some students, particularly those who were older at baseline and no longer eligible to participate at follow-up, post-pandemic, this also provided a more accurate representation of potential bias from student-level follow-up nonresponse.

Second, we utilized self-reported measures of both alcohol use and change in screen time, and therefore responses may have been subject to social desirability bias. Third, measures used to assess perceived changes in adolescents’ screen time behaviours during the pandemic have not been validated. Fourth, while we utilized longitudinal data to examine change in behaviours over time, we examined change in screen time and change in alcohol use concurrently. As a result, we cannot confirm whether changes in screen time were affected by changes in alcohol use. However, research suggests that the predominant direction of effect is from screen time to alcohol use or substance use.^2^,^5^,^46^,^48^

While associations between socioeconomic status and youths’ screen time and alcohol use have been previously noted,^4^,^15^ because the COMPASS study does not collect data on household income and nearly one-quarter of the sample had missing responses for weekly spending money or part-time employment, we did not include this proxy measure in our analyses.

Finally, given the small sample sizes of participants who did not use alcohol and of those who did at the different time points, we were unable to explore whether these associations differed by sex or age at pandemic onset or among those with pre-pandemic elevated screen time use. Because of the complexity of the adjusted models, accounting for school-level clustering led to convergence issues in the multilevel models. Consequently, the precision of the effects in these models is overstated. However, we observed minimal clustering at the school level in the models we were able to run.

## Conclusion

This study contributes to the current evidence documenting the link between adolescents’ screen time and social media use with alcohol use by identifying that these relationships persisted during the COVID-19 pandemic. Existing large-scale population-based studies should continue to explore the long-term relationships between screen time and alcohol use in adolescence and into early adulthood. Given how the digital landscape has been expanding in recent years, it is important to investigate if specific groups of adolescents are at heightened risk, taking into account factors such as their age during the pandemic, their sex and gender, and any pre-existing mental health conditions. Understanding why increased screen time is linked to subsequent alcohol use, and identifying potential at-risk groups, would help in developing effective prevention strategies that reduce underage drinking. This includes implementing tighter regulations on alcohol-related social media content and providing targeted mental health support.

## Acknowledgements

The COMPASS study has been supported by a bridge grant from the Canadian Institutes of Health Research (CIHR) Institute of Nutrition, Metabolism and Diabetes through the “Obesity – Interventions to Prevent or Treat” priority funding awards (OOP-110788, awarded to STL); an operating grant from the CIHR Institute of Population and Public Health (IPPH) (MOP-114875, awarded to STL); a CIHR project grant (PJT-148562, awarded to STL); a CIHR bridge grant (PJT-149092, awarded to KAP/STL); a CIHR project grant (PJT-159693, awarded to KAP); a research funding arrangement with Health Canada (#1617-HQ-000012, contract awarded to STL);aCIHR–Canadian Centre on Substance Use and Addiction team grant (OF7 B1-PCPEGT 410-10-9633, awarded to STL); and a project grant from the CIHR IPPH (PJT-180262, awarded to STL and KAP). The COMPASS-Quebec project also benefits from funding from the Ministre de la sant et des services sociaux of the province of Quebec and the Direction rgionale de sant publique du CIUSSS de la Capitale-Nationale. KAP is the Tier II Canada Research Chair in Child Health Equity and Inclusion. TV is funded by the Public Health Agency of Canada through the Federal Student Work Experience Program.

## Conflicts of interest

At the time of article submission, STL was one of the *HPCDP* Journal’s Associate Scientific Editors. He recused himself from the review process for this article.

The authors declare that they have no conflicts of interest.

## Authors’ contributions and statement

TV: Formal analysis, project administration, writing—original draft, writing—review and editing.

CO: Conceptualization, methodology, project administration, formal analysis, writing—original draft, writing—review and editing.

EC: Conceptualization, methodology, project administration, writing—review and editing.

AA: Writing—review and editing.

SUK: Writing—review and editing.

KB: Writing—review and editing.

STL: Investigation, data curation, funding acquisition, project administration, resources, writing—review and editing.

RE: Conceptualization, methodology, project administration, supervision, writing—review and editing.

The content and views expressed in this article are those of the authors and do not necessarily reflect those of the Government of Canada.

## References

[B01] Sigmund E (2021). Weekday-weekend sedentary behavior and recreational screen time patterns in families with preschoolers, schoolchildren, and adolescents: cross-sectional three cohort study. Int J Environ Res Public Health.

[B02] Carson V, Pickett W, Janssen I (2011). Screen time and risk behaviors in 10-to 16-year-old Canadian youth. Prev Med.

[B03] Sweetser P, Johnson D, Ozdowska A, Wyeth P (2012). Active versus passive screen time for young children. Australas J Early Child.

[B04] Toombs E, Mushquash CJ, Mah L, Short K, Young NL, Cheng C, et al (2022). Increased screen time for children and youth during the COVID-19 pandemic [Internet]. Toombs E, Mushquash CJ, Mah L, Short K, Young NL, Cheng C, et al.

[B05] Trott M, Driscoll R, Irlado E, Pardhan S (2022). Changes and correlates of screen time in adults and children during the COVID-19 pandemic: a systematic review and meta-analysis. EClinicalMedicine.

[B06] Vogels EA, Gelles-Watnick R, Massarat N Teens, social media and technology 2022 [Internet]. Pew Research Center.

[B07] Children (5 11 years) and youth (12 17 years): Canadian 24-hour movement guidelines for the children and youth (5 17 years): an integration of physical activity, sedentary behaviour, and sleep [Internet]. an integration of physical activity, sedentary behaviour, and sleep [Internet]. Ottawa (ON): CSEP.

[B08] Riehm KE, Feder KA, Tormohlen KN, Crum RM, Young AS, Green KM, et al (2019). Associations between time spent using social media and internalizing and externalizing problems among US youth. JAMA Psychiatry.

[B09] Physical activity, sedentary behaviour and sleep (PASS) indicators: data tool [Internet]. Centre for Surveillance and Applied Research.

[B10] Leatherdale ST, Ahmed R (2011). Screen-based sedentary behaviours among a nationally representative sample of youth: are Canadian kids couch potatoes. Chronic Dis Inj Can.

[B11] Shoshani A, Kor A, Farbstein-Yavin S, Gvion Y (2024). Risk and protective factors for substance use and media addictive behaviors in adolescents during the COVID-19 pandemic. J Adolesc.

[B12] Kovacs VA, Starc G, Brandes M, Kaj M, Blagus R, ek B, et al (2022). Physical activity, screen time and the COVID-19 school closures in Europe an observational study in 10 countries. Eur J Sport Sci.

[B13] Li X, Vanderloo LM, Keown-Stoneman CD, Cost KT, Charach A, Maguire JL, et al (2021). Screen use and mental health symptoms in Canadian children and youth during the COVID-19 pandemic. JAMA Netw Open.

[B14] Shoshani A, Kor A (2024). The longitudinal impact of the COVID-19 pandemic on adolescents internalizing symptoms, substance use, and digital media use. Eur Child Adolesc Psychiatry.

[B15] Toigo S, Betancourt MT, Prince SA, Colley RC, Roberts KC (2024). Sociodemographic differences in recreational screen time before and during the COVID-19 pandemic in Canada. Health Rep.

[B16] zina-Im LA, Beaulieu D, Turcotte S, Roussel-Ouellet J, Bouchard D (2022). Association between recreational screen time and sleep quality among adolescents during the third wave of the COVID-19 pandemic in Canada. Int J Environ Res Public Health.

[B17] Mitra R, Moore SA, Gillespie M, Faulkner G, Vanderloo LM, Chulak-Bozzer T, et al (2020). Healthy movement behaviours in children and youth during the COVID-19 pandemic: exploring the role of the neighbourhood environment. Health Place.

[B18] Moore SA, Faulkner G, Rhodes RE, Brussoni M, Chulak-Bozzer T, Ferguson LJ, et al (2020). Impact of the COVID-19 virus outbreak on movement and play behaviours of Canadian children and youth: a national survey. Int J Behav Nutr Phys Act.

[B19] Moore SA, Faulkner G, Rhodes RE, Vanderloo LM, Ferguson LJ, Guerrero MD, et al (2021). Few Canadian children and youth were meeting the 24-hour movement behaviour guidelines 6-months into the COVID-19 pandemic: follow-up from a national study. Appl Physiol Nutr Metab.

[B20] Zhu X, Griffiths H, Xiao Z, Ribeaud D, Eisner M, Yang Y, et al (2023). Trajectories of screen time across adolescence and their associations with adulthood mental health and behavioral outcomes. J Youth Adolesc.

[B21] Nagata JM, Memon Z, Talebloo J, Li K, Low P, Shao IY, et al (2025). Prevalence and patterns of social media use in early adolescents. Acad Pediatr.

[B22] Nagata JM, Cortez CA, Cattle CJ, Ganson KT, Iyer P, Bibbins-Domingo K, Baker FC (2022). Screen time use among US adolescents during the COVID-19 pandemic: findings from the Adolescent Brain Cognitive Development (ABCD) Study. JAMA Pediatr.

[B23] Coyne SM, Padilla-Walker LM, Holmgren HG (2018). A six-year longitudinal study of texting trajectories during adolescence. Child Dev.

[B24] Wiciak MT, Shazley O, Santhosh D (2022). An observational report of screen time use among young adults (ages 18-28 years) during the COVID-19 pandemic and correlations with mental health and wellness: international, online, cross-sectional study. JMIR Form Res.

[B25] Mougharbel F, Chaput JP, Sampasa-Kanyinga H, Hamilton HA, Colman I, Leatherdale ST, et al (2023). Heavy social media use and psychological distress among adolescents: the moderating role of sex, age, and parental support. Front Public Health.

[B26] Su H, Lyu D, Huang K, Yan J (2024). Association of physical activity, screen time and sleep with substance use in children and adolescents: a large sample cross-sectional study. Front Public Health.

[B27] Tebar WR, Christofaro DG, Diniz TA, Lofrano-Prado MC, Botero JP, Correia MA, et al (2021). Increased screen time is associated with alcohol desire and sweetened foods consumption during the COVID-19 pandemic. Front Nutr.

[B28] Kelleghan AR, Leventhal AM, Cruz TB, Bello MS, Liu F, Unger JB, et al (2020). Digital media use and subsequent cannabis and tobacco product use initiation among adolescents. Drug Alcohol Depend.

[B29] Nagata JM, Shim J, Low P, Ganson KT, Testa A, He J, et al (2025). Prospective association between screen use modalities and substance use experimentation in early adolescents. Drug Alcohol Depend.

[B30] Riehm KE, Thrul J, Barrington-Trimis JL, Kelleghan A, Mojtabai R, Leventhal AM (2021). Prospective association of digital media use with alcohol use initiation and progression among adolescents. Alcohol Clin Exp Res.

[B31] Sampasa-Kanyinga H, Chaput JP (2016). Use of social networking sites and alcohol consumption among adolescents. Public Health.

[B32] Vandenbroucke JP, Elm E, Altman DG, tzsche PC, Mulrow CD, Pocock SJ, et al (2014). Strengthening the reporting of observational studies in epidemiology (STROBE): explanation and elaboration. Int J Surg.

[B33] Leatherdale ST, Brown KS, Carson V, Childs RA, Dubin JA, Elliott SJ, et al (2014). The COMPASS study: a longitudinal hierarchical research platform for evaluating natural experiments related to changes in school-level programs, policies and built environment resources. BMC Public Health.

[B34] Reel B, Battista K, Leatherdale ST COMPASS protocol changes and recruitment for online survey implementation during the COVID-19 pandemic [Internet]. University of Waterloo.

[B35] Gohari MR, Varatharajan T, Patte KA, MacKillop J, Leatherdale ST (2023). The intersection of internalizing symptoms and alcohol use during the COVID-19 pandemic: a prospective cohort study. Prev Med.

[B36] Lee EY, Hunter S, Leatherdale ST, Carson V (2019). Sociodemographic correlates of physical activity and screen time among adolescents in Canada and Guatemala: results from the COMPASS system. Glob Health Promot.

[B37] Romano I, Ferro MA, Patte KA, Leatherdale ST (2022). Measurement invariance of the GAD-7 and CESD-R-10 among adolescents in Canada. J Pediatr Psychol.

[B38] Spitzer RL, Kroenke K, Williams JB, we B (2006). A brief measure for assessing generalized anxiety disorder: the GAD-7. A brief measure for assessing generalized anxiety disorder: the GAD-7. Arch Intern Med.

[B39] Elton-Marshall T, Leatherdale ST, Manske SR, Wong K, Ahmed R, Burkhalter R (2011). Research methods of the Youth Smoking Survey (YSS). Chronic Dis Inj Can.

[B40] Leatherdale ST, Laxer RE, Faulkner G (2014). Reliability and validity of the physical activity and sedentary behaviour measures in the COMPASS study [Internet]. University of Waterloo.

[B41] Wong SL, Leatherdale ST (2009). Association between sedentary behavior, physical activity, and obesity: inactivity among active kids. Prev Chronic Dis.

[B42] Newman DA (2014). Missing data: five practical guidelines. Organ Res Methods.

[B43] multinom: Fit multinomial log-linear models [software]. nnet (version 7.

[B44] Boers E, Afzali MH, Conrod P (2020). A longitudinal study on the relationship between screen time and adolescent alcohol use: the mediating role of social norms. Prev Med.

[B45] Marciano L, Camerini AL, Morese R (2021). The developing brain in the digital era: a scoping review of structural and functional correlates of screen time in adolescence. Front Psychol.

[B46] Christodoulou G, Majmundar A, Chou CP, Pentz MA (2020). Anhedonia, screen time, and substance use in early adolescents: a longitudinal mediation analysis. J Adolesc.

[B47] Smit K, Voogt C, Otten R, Kleinjan M, Kuntsche E (2022). Why adolescents engage in early alcohol use: a study of drinking motives. Exp Clin Psychopharmacol.

[B48] Gardner LA, Debenham J, Newton NC, Chapman C, Wylie FE, Osman B, et al (2022). Lifestyle risk behaviours among adolescents: a two-year longitudinal study of the impact of the COVID-19 pandemic. BMJ Open.

